# Chang’E-5 samples reveal high water content in lunar minerals

**DOI:** 10.1038/s41467-022-33095-1

**Published:** 2022-09-10

**Authors:** Chuanjiao Zhou, Hong Tang, Xiongyao Li, Xiaojia Zeng, Bing Mo, Wen Yu, Yanxue Wu, Xiandi Zeng, Jianzhong Liu, Yuanyun Wen

**Affiliations:** 1 Center for Lunar and Planetary Sciences, Institute of Geochemistry, Chinese Academy of Sciences, 550081 Guiyang, China; 2 College of Earth and Planetary Sciences, University of Chinese Academy of Sciences, 100049 Beijing, China; 3 CAS Center for Excellence in Comparative Planetology, 230026 Hefei, China; 4 Key Laboratory of Space Manufacturing Technology, Chinese Academy of Sciences, 100094 Beijing, China; 5 Analysis and Test Center, Guangdong University of Technology, 510006 Guangzhou, China

**Keywords:** Mineralogy, Geochemistry, Rings and moons

## Abstract

The formation and distribution of lunar surficial water remains ambiguous. Here, we show the prominence of water (OH/H_2_O) attributed to solar wind implantation on the uppermost surface of olivine, plagioclase, and pyroxene grains from Chang’E-5 samples. The results of spectral and microstructural analyses indicate that solar wind-derived water is affected by exposure time, crystal structure, and mineral composition. Our estimate of a minimum of 170 ppm water content in lunar soils in the Chang’E-5 region is consistent with that reported by the Moon Minerology Mapper and Chang’E-5 lander. By comparing with remote sensing data and through lunar soil maturity analysis, the amount of water in Chang’E-5 provides a reference for the distribution of surficial water in middle latitude of the Moon. We conclude that minerals in lunar soils are important reservoirs of water, and formation and retention of water originating from solar wind occurs on airless bodies.

## Abstract (web summary)

Lunar soils returned by China’s Chang’E−5 (CE5) mission record the unique information of solar wind essential to understanding the preservation and distribution of lunar surficial water. Here the authors report abundant water formed by solar wind implantation in minerals of CE5 lunar soils; the water content in CE5 lunar soils is estimated to be ~ 170 ppm.

## Introduction

Infrared reflectance spectra obtained by means of remote sensing by Cassini, Deep Impact, and Chandrayaan-1 revealed a widespread presence of solar wind-derived OH/H_2_O on the lunar surface^1–4^. Variations in temperature on diurnal timescales as well as composition and maturity of lunar soils are the main factors influencing the lunar surficial OH/H_2_O cycle^1,5–7^. Direct analysis of water content and hydrogen isotopes in Apollo samples has provided strong evidence that solar wind proton implantation is an important source of water on the lunar surface^8–10^. The occurrence of the implanted proton from solar wind in silicate minerals and glass is various and controversial. These could be retained as OH, even H_2_O, in the structure or in the form of H in the defects. In this study, the presence of all the H-bearing species, such as OH, H_2_O, and H, is referred to simply as “water.” Previous studies on Apollo lunar soils have reported the presence of solar wind-derived water only in agglutinitic glass, volcanic glass, and plagioclase, indicating a heterogenous presence of water in different lunar grains. However, the main silicate minerals on the lunar surface, including olivine, pyroxene, and plagioclase, have been verified to produce water through H^+^ implantation^11–13^. Simulation experiments have confirmed that the efficiency of water formation by the solar wind differs based on parameters such as exposure time and characteristics of lunar grains (e.g., composition and structure)^14,15^. A comprehensive understanding of the formation of water by solar wind implantation in different lunar minerals and assessment of the distribution of solar wind-derived water on the lunar surface has not yet been well investigated.

China’s Chang’E-5 (CE5) mission returned 1.731 kg of lunar materials from Northeastern Oceanus Procellarum basin (43.06°N, 51.92°W) of the Moon, which is located at a higher latitude than that explored by all previous sampling missions^16,17^. Radiometric dating using the lead-lead (Pb-Pb) isotope isochron technique revealed the age of CE5 lunar samples to be 2030 ± 4 million years, which is much younger than that of lunar samples collected by the Apollo and Luna missions^18^. Lunar soil contains water from three types of sources: water originating from the lunar interior, solar wind, and comets and meteoroids^19–21^. A study based on analysis of water in apatite and melt inclusions from CE5 samples reported the presence of a dry lunar mantle reservoir for CE5 basalts^22^. Considering the exposure of CE5 samples to the lunar surface, the lunar grains are expected to record the information of solar wind implantation and form solar wind-derived water. The unique sampling location and age of CE5 samples, different from Apollo samples, are expected to provide a reference for the preservation and distribution of surficial water on the Moon.

Pyroxene, plagioclase, and olivine, the primary minerals in the CE5 lunar soil, are likely to be the main reservoirs of solar wind-derived water^23^. Our investigation of solar wind-derived water in lunar minerals based on analyzing pyroxene, plagioclase, and olivine grains selected from CE5 lunar samples using Fourier transform infrared spectroscopy (FTIR), nanoscale secondary ion mass spectrometry (NanoSIMS), and transmission electron microscopy (TEM) identifies the water in these minerals to be formed solely via solar wind implantation without the influence of meteorites and micrometeorites. The content and occurrence of solar wind-derived water in different lunar minerals are determined. The effect of composition and structure, as well as the maturity (i.e., exposure times to solar wind) of the minerals, are constrained. This study has important implications for understanding the evolution of water on the lunar surface and evaluating the contribution of solar wind protons to the water reservoirs of the lunar surface.

## Results and discussion

### Water content and hydrogen isotope ratio

**Fig. 1:**
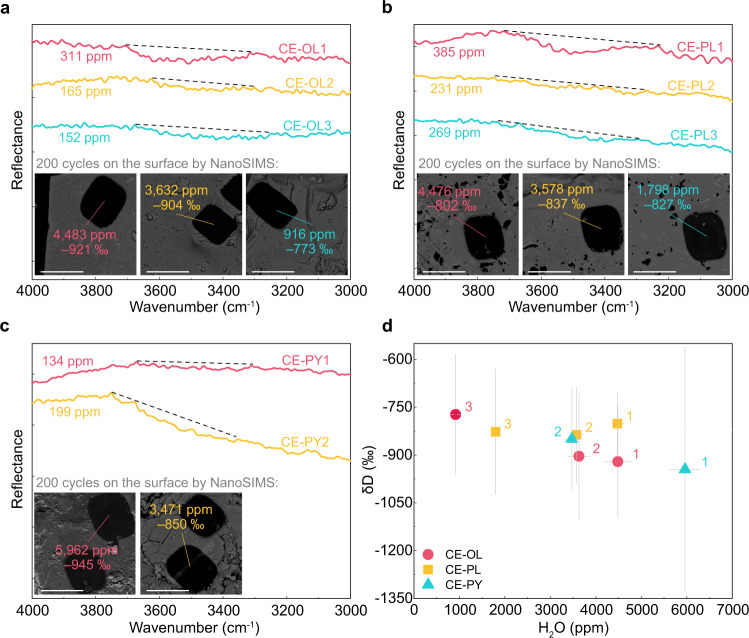
Water content and hydrogen isotope composition of lunar grains. **a**–**c** Reflectance infrared spectra of **a** olivine, **b** plagioclase, and **c** pyroxene. Points of analysis for NanoSIMS measurements are marked on the backscattered electron images (each point of analysis is marked with the same color as that used for depicting the spectra and the numbers correspond to the water content and δD). The scale bar is 10 nm. The solid lines are smoothed spectra obtained using the Fast Fourier Transform algorithm, and different colors of the solid lines represent different samples; the dashed lines indicate the baseline reflectance of absorption peaks. **d** δD versus H_2_O plots of lunar minerals. δD = {[(D/H)_sample_/(D/H)_VSMOW_] − 1} × 1000; VSMOW Vienna standard mean ocean water^19^. The round dots represent δD and water content of olivine, the square dots represent that of plagioclase, and the triangular dots represent that of pyroxene. The error bars (gray line) represent 2σ. The difference in water content from reflection infrared and NanoSIMS is mainly attributed to the difference in measurement depth; for details, see Methods.

Reflectance infrared spectra of all the minerals from CE5 lunar samples exhibit broad absorption in the range 3200–3800 cm^−1^ and centered around 3500 cm^−1^, which indicates the existence of OH groups (Fig. 1a–c, Supplementary Data 1). In particular, a small peak centered around 3300 cm^−1^ and a broad peak centered around 1640 cm^−1^ in CE-PL2 sample most likely represent the presence of H_2_O (Fig. 1b, Supplementary Fig. 1)^24,25^. The water content is determined to be 152 ± 14 to 311 ± 30 ppm H_2_O in olivine (CE-OL1, CE-OL2, CE-OL3), 231 ± 16 to 385 ± 27 ppm H_2_O in plagioclase (CE-PL1, CE-PL2, CE-PL3), and 134 ± 19 to 199 ± 28 ppm H_2_O in pyroxene (CE-PY1, CE-PY2) based on corrections implemented with reference to calibration lines derived from analysis of the corresponding terrestrial minerals (Supplementary Table 1, Supplementary Fig. 2, Methods section). NanoSIMS measurements show that the uppermost surfaces of these minerals (~200 nm) are clearly H-rich and D-poor (Fig. 1a–c, Supplementary Table 2, Supplementary Data 2). The equivalent water contents of the surfaces of the olivine, plagioclase, and pyroxene samples are 916 ± 38 to 4483 ± 314 ppm, 1798 ± 81 to 4476 ± 142 ppm, and 3471 ± 166 to 5962 ± 335 ppm, respectively. A considerable difference in water content on the surface and in the bulk grain, as determined using NanoSIMS and FTIR analyses, respectively, implies that water is concentrated in the uppermost layer of these grains. The hydrogen isotope ratio (expressed as δD) of all minerals ranged from –773 ± 188 to –945 ± 384‰, which is close to that of the solar wind (δD ≈ –1000‰) (Fig. 1d, Supplementary Table 2). Given that cosmic-ray spallation can produce D in lunar grains, the measured δD values represent the upper limit of the initial value of water in the samples; however, the production of D by cosmic-ray spallation is negligible^26,27^. Taking together the very low δD values and the fact that water in minerals of CE5 lunar samples is concentrated on the uppermost surface, we conclude that the water measured in our analysis is derived from solar wind implantation. This solar wind-derived water exists mostly in the form of OH and possibly as H_2_O in some plagioclase grains.

### The comparison of microstructural characteristics and depth distribution of solar wind-derived water in the uppermost surface

**Fig. 2:**
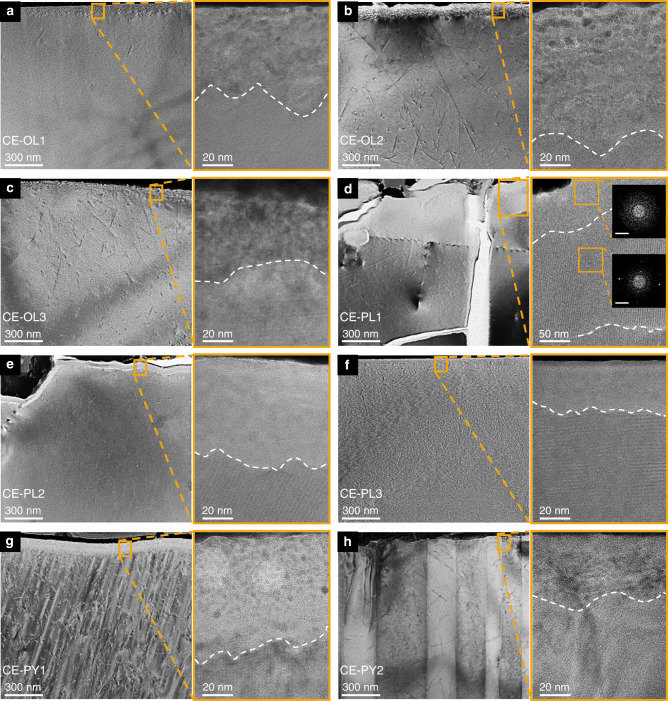
Characteristics of lunar grain surfaces. **a**–**c** TEM bright-field images of olivine samples **a** CE-OL1, **b** CE-OL2, and **c** CE-OL3. **d**–**f** TEM bright-field images of plagioclase samples **d** CE-PL1, **e** CE-PL2, and **f** CE-PL3. **g**, **h** TEM bright-field images of pyroxene samples **g** CE-PY1 and **h** CE-PY2. For each group of images, the image on the right is a magnified view of the orange box with the dashed border in the left panel; the scale bar of the left image is 300 nm, and the scale bar of the right image is 20 nm, except for (**d**) for which the scale of the right image is 50 nm. In the right panel of (**d**), the two images on the right are selected area electron diffraction (SAED) patterns of the orange rectangular regions, where the top image indicates complete amorphization and the bottom image indicates partial amorphization. The scale bar of SAED patterns is 2 nm^−1^. The degree of amorphous region in the minerals was as follows: CE-OL3 ≈ CE-OL1 < CE-OL2, CE-PL3 < CE-PL2 < CE-PL1, CE-PY2 < CE-PY1. The order of values of track density in the minerals is as follows: CE-OL1 < CE-OL3 < CE-OL2, CE-PL3 < CE-PL2 < CE-PL1, CE-PY2 < CE-PY1.

The TEM images reveal partially and/or completely amorphous rims on the uppermost surfaces of the olivine, plagioclase, and pyroxene samples (Fig. 2a–h). The composition of these amorphous rims is consistent with that of the underlying crystal, thus serving as evidence for solar wind implantation-induced damage (Supplementary Figs. 3–5, Methods section). The rims of the olivine grains show a highly defective crystalline (40–100 nm thick) structure without complete amorphization (Fig. 2a–c). All plagioclase grain rims were completely amorphous, with a thickness of 40–100 nm (Fig. 2d–f). Plagioclase with the highest water content is observed to have a partially amorphous layer below the completely amorphous layer, with a thickness of up to 400 nm (Fig. 2d). In the pyroxene samples, the damaged rim of one grain is completely amorphous, and that of another grain is partially amorphous (Fig. 2g, h). The differences in the degree of amorphousness among different mineral types indicate that the compact crystal structure of olivine allows it to resist the destructive effects of solar wind particles, while plagioclase is vulnerable to damage by solar wind^28^. Nanophase Fe metal (npFe^0^) grains are dispersed as inclusions in the damaged rims of Fe-rich pyroxene and olivine. This indicates that solar wind implantation may contribute to the formation of npFe^0^ on the uppermost surface of lunar grains, which needs further investigation^14,29–31^. The microstructural features, together with the water content and δD value of the surface of these minerals, suggest the importance of solar wind implantation and the complexity of the conditions of water formation.

**Fig. 3:**
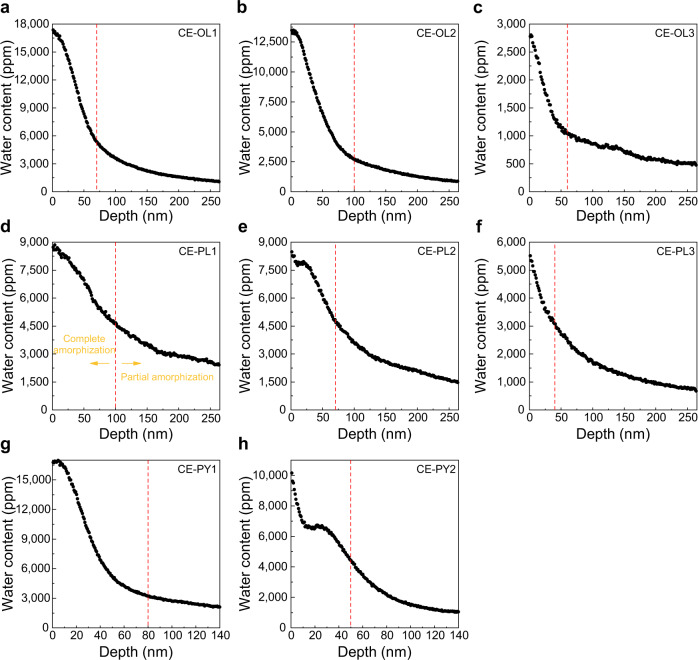
Depth distribution characteristics of water in lunar minerals. **a**–**c** Depth profiles of water content in olivine samples **a** CE-OL1, **b** CE-OL2, and **c** CE-OL3. **d**–**f** Depth profiles of water content in plagioclase samples **d** CE-PL1, **e** CE-PL2, and **f** CE-PL3. **g**, **h** Depth profiles of water content in pyroxene samples **g** CE-PY1 and **h** CE-PY2. The depths of the NanoSIMS measurements were obtained using the thin slices of analysis regions prepared by the FIB-SEM system. The red dashed line denotes the maximum thickness of the damaged rims of the grains for samples (**a**–**c**), (**e**–**g**), while for sample (**d**), it represents the boundaries of the complete and partial amorphous rims, which is observed by the TEM.

To understand the depth distribution of solar wind-derived water in lunar soils, the depth profiles of water content in minerals are plotted by combining the results of NanoSIMS and TEM measurements (Fig. 3a–h). Generally, all profiles show that the water content is the highest at the outermost surface of minerals and decreases with an increase in depth. The content of water decreases sharply in the damaged rims, while it decreases slowly in the underlying crystals. The results reveal that the water formed by the solar wind is mainly concentrated in the amorphous rims, and a small amount of solar wind-derived water can still be preserved in the underlying crystal. This indicates that the implanted H^+^ of solar wind in minerals could diffuse into the crystals underlying the damaged rims. From the profile of water content, we can predict that the deeper region of the surface (>200 nm) still has a certain amount of solar wind-derived water.

### Primary influences on the formation of solar wind-derived water

Overturning of lunar soil results in lunar grains having different exposure times to solar wind. The exposure time directly affects the amount of solar wind-derived water present on the surface of minerals^14^. As solar wind particle implantation can cause the amorphization of the mineral surface, the degree of the amorphous region in the grain surface can be used to assess the exposure time of lunar grains^32,33^. Generally, the longer the implanted time of solar wind, the higher the degree of amorphization on the surface of minerals, that is, the thicker the amorphous width and the higher the grade of amorphization. The TEM images obtained in our study show that for the same type of minerals, the thickness and degree of amorphization of damaged rims are generally positively correlated with water content (Fig. 2a–h). This indicates that the water formed via solar wind H^+^ implantation strongly depends on exposure time, although the minerals examined in this study were not saturated by solar wind implantation through the investigation of the damage rims in Apollo samples and simulation experiment results^12,33^. This conclusion is further supported by the detection of radiation tracks in the host minerals, that is, the order of values of track density is approximately consistent with the degree of the amorphous region in the minerals. The radiation tracks produced by the solar energetic particles (SEPs) can penetrate lunar soils at millimeter depths and leave tracks in the crystals. Although impact gardening on the lunar surface would cause the grains to be buried and still receive radiation from SEPs, previous studies have shown that the rate of track accumulation attenuates strongly when grain is buried at any depth, which indicates a low accumulation of radiation tracks after the grains are buried^28^. A positive correlation between water content and track density can be observed in these grains, suggesting that the track density can be used to reflect the exposure time when the grains receive solar wind implantation. It can be inferred that the content of solar wind-derived water in the grains is directly proportional to the exposure time. Based on the water content on the surface (~200 nm) of lunar minerals, we estimate the accumulated implantation time of solar wind protons for CE5 lunar soils to be about 270–4212 years (Methods Sections).

Observations for CE-OL1 yield an exception to the above statements. NanoSIMS measurements show it to have the highest water content; however, TEM reveals only a few radiation tracks to be present in CE-OL1 grains (Fig. 2a). Therefore, reasons other than exposure time must be considered to account for the differences in solar wind implantation for the case of CE-OL1 grains. Differences in chemical composition can influence solar wind implantation in the same type of minerals. The surface of Fe-rich olivine is more easily damaged by the solar wind than that of Mg-rich olivine, and the content of solar wind-derived water cannot be compared solely based on radiation tracks and the degree of amorphousness of the surface. A study by Wang and Ewing based on krypton ion irradiation experiments on a series of olivine with five members revealed that Fe-rich olivine is more susceptible to ion damage owing to its lower melting temperature and a higher percentage of covalent bonds^34^. In our study, the Fe/Mg ratios in olivine grains analyzed using energy-dispersive X-ray spectrometry (EDS) are found to differ among samples (Supplementary Table 2). The CE-OL1 grain is relatively Mg-rich with an Fo value (100 × Mg/[Mg + Fe] molar ratio) of 61, whereas the other two grains are relatively Fe-rich with Fo values less than 50. The crystal structure of the Mg-rich olivine (CE-OL1) with the same exposure time shows minimal damage to rims and tracks resulting from irradiation by solar wind and solar flares.

**Fig. 4:**
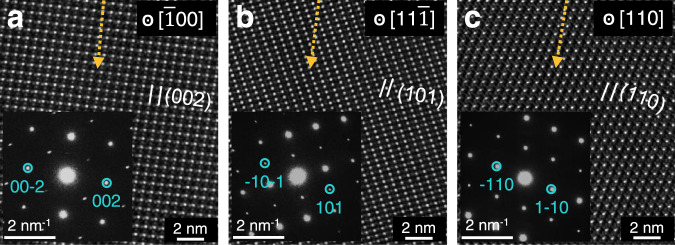
Crystal orientation of plagioclase grains implanted by solar wind particles. **a**–**c** High-resolution (HR) TEM images and corresponding selected area electron diffraction (SAED) patterns depicting residual crystal planes along the [−100], [11-1], and [110] zone-axis for the plagioclase samples **a** CE-PL1, **b** CE-PL2, and **c** CE-PL3, respectively. The scale bar of HR-TEM images is 2 nm, and the scale bar of SAED patterns is 2 nm^−1^. The orange arrows represent the approximate implantation direction of solar wind particles.

The plagioclase grains in our study show a similar chemical composition with An values (100 × Ca/[Ca + Na + K] molar ratio) of ~80, but the characteristics of damage rims varied considerably between CE-PL1 and CE-PL2, which have small differences in water content and exposure time (Supplementary Table 2). Previous studies have shown that the maximum depth of structural damage caused by solar wind particles on plagioclase surfaces is ~200 nm^28^. However, the maximum depth of the damaged rim in CE-PL1, including the completely and partially amorphous parts, reached ~500 nm in the samples examined in our study (Fig. 2d). The effect of exposure time alone could not possibly result in such a large difference in structural damage. The differences in the crystal orientation of solar wind-implanted particles could potentially explain the large difference in structural damage^35,36^. Selected area electron diffraction (SAED) and high-resolution (HR) TEM images identify the crystal planes in the three plagioclase grains (Fig. 4a–c). The implantation direction of solar wind particles is found to be approximately along the *c*-axis of the CE-PL1 crystal, which is parallel to the perfect cleavage systems. The orientation parallels the cleavage, results in the weakest bonding forces among atoms, and renders the grains most vulnerable to damage by solar wind particles. We infer that solar wind particles implanted into the plagioclase at high fluence along the direction of cleavage led to the production of a thick damaged rim in CE-PL1. The analyses of the crystal orientation in plagioclase grains provide evidence of the solar wind-derived damage rim having a thickness of more than ~500 nm, which is consistent with values from simulations reported in previous studies based on the Stopping and Range of Ions in Matter package^33^.

### Implications of the contribution of solar wind implantation to water content on the lunar surface

Analysis of solar wind-derived water in the main minerals in lunar grains, including olivine, pyroxene, and plagioclase, can facilitate estimation of the total amount of water stored in lunar soils in the CE5 region and evaluation of the contribution of the solar wind-derived water toward constituting the total water content. In our study, the amount of water stored in CE5 lunar soils is estimated based on the reflectance infrared spectra of the mineral grains. Assuming that all the water formed via solar wind is present in olivine, plagioclase, and pyroxene and accounting for the mineral abundance of CE5 lunar soils, we calculate the average water content in CE5 lunar soils to be ~170 ppm (Methods Section). CE5 samples contain a certain amount of agglutinitic glass, which is also an important host material for solar wind-derived water. Consequently, the estimated water content of 170 ppm is considered lower than the actual water content in CE5 lunar soils. This value is similar to that obtained from observations by the Moon Mineralogy Mapper (M3) onboard the Chandrayaan-1 and the highest values from Lunar Mineralogical Spectrometer onboard the Chang’E-5 lander^6,37,38^. Note that Liu et al. consider^38^ such a high water content to reflect the contribution of indigenous water in apatite, while the contribution of solar wind is very low. However, our analysis shows that minerals with water of hundreds of ppm in the lunar soils include olivine, plagioclase, and pyroxene, and the δD values demonstrate that the water is derived from the solar wind. Thus, we conclude that solar wind implantation can contribute abundant water to the lunar soils in the CE5 region. The contribution of solar wind-derived water to the total water content of the lunar surface is larger than that of water derived from the lunar interior, which exists mainly in apatite and melts inclusions^22^. Therefore, we consider that solar wind-derived water may play an important role in the future utilization of lunar water. In addition, similar mechanisms resulting in the formation of water via solar wind implantation may be common on airless bodies, such as Mercury and asteroids, throughout the solar system.

To further evaluate the distribution of water formed via the solar wind, the effective single particle absorption thickness (ESPAT) values that are frequently used in remote sensing to quantify water on the lunar surface are calculated from the reflection infrared spectra of mineral grains (Methods Section)^6,37,39,40^. The ESPAT values at the wavelength of 2.85–3 µm of the mineral grains are estimated to range from 0.028 to 0.081, with an average value of 0.051, which are generally higher than those derived from M3 by Li and Milliken (Supplementary Table 3)^6^. Previous studies have shown that the ESPAT values at the wavelength of 2.85–3 µm exhibit a linear correlation with water content with different slopes, which depends on the size of grain^6^. Based on this, the corresponding water content of each mineral calculated by multiplying the slopes is 88–274 ppm (Supplementary Table 3). According to the ESPAT-derived water content of grains and mineral abundance in the CE5 region, we estimate the water content in CE5 lunar soil to be at least 146 ppm. This value is consistent with that calculated by the laboratory calibration method (~170 ppm), given the error of ~20% in the water content estimated from the ESPAT value.

**Fig. 5:**
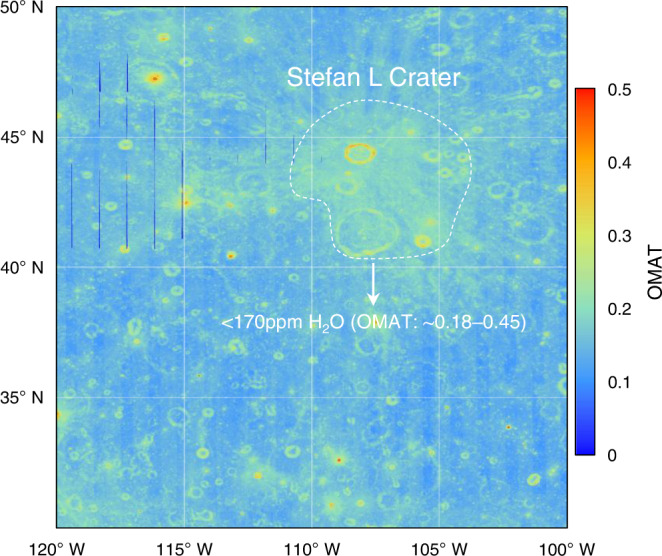
Optical maturity (OMAT) image for Stefan L crater and its surrounding areas. The OMAT values were derived from Lemelin et al.^44^. The dashed area indicates the Stefan L Crater with larger OMAT values.

As a parameter reflecting the degree of space weathering of lunar soils, optical maturity (OMAT) has been demonstrated to correlate with water content in regions with latitudes higher than 30° ^6,41^. At mid-latitudes, the analysis of the maturity in the CE5 region can provide a reference for the water content in the lunar soil. This is because the distribution of solar wind-derived water was severely constrained by temperature on the lunar surface. At low latitudes (<30°), the maximum temperature of the lunar surface can exceed 400 K, and the water in lunar soils is thought to escape to some extent. The relationship between maturity and water content at low latitudes is weak. At latitudes higher than 30°, because the highest temperature is lower than that at low latitudes, the maturity of lunar soils significantly affects the formation of solar wind-derived water. The sampling site of the CE5 mission is located at the longitude and latitude of 51.916°W and 43.058°N, where the highest temperature is ~350 K^42^. The OMAT values of CE5 regions range from 0.17 to 0.22, which suggests a relatively immature lunar soil^43^. Our results show the ~170 ppm content of solar wind-derived water in the CE5 region with relatively low maturity. Remote sensing data have revealed a similar correlation between water content and maturity when there is the sufficient water content at latitudes of 30°N to 60°N. That is, the content of solar wind-derived water increases with the maturity of the lunar soils. A similar range of OMAT values at the CE5 region can be found to the north of the Oceanus Procellarum and Imbrium basin, which implies that these regions may contain an approximate amount of water (~170 ppm)^44^. In the highlands on the northwest side of Oceanus Procellarum, the lunar soils are mature with the OMAT values generally <0.15 and can be expected to be abundant in water. Meanwhile, the young craters and their rays in the highlands are immature, with a lower expected water content than that in the surrounding areas. This can be supported by the global distribution of lunar surface water derived by Li and Milliken, where the Stefan L crater and its rays (44.6°N, 107.7°W) with OMAT values generally larger than 0.18 exhibit significantly lower water content than the surrounding areas (Fig. 5)^6^.

## Methods

### Sample preparation

The Chang’E-5 (CE5) lunar samples (CE5C0400YJFM00505) used in this study were scooped from the lunar surface and allocated by China National Space Administration. These samples were packaged in the ultraclean room at Extraterrestrial Sample Curation Center of National Astronomical Observatories, Chinese Academy of Sciences. The CE5 samples were sealed in nitrogen and transferred to a glovebox with flowing high-purity argon at the Institute of Geochemistry, Chinese Academy of Sciences. Eighty-five grains of lunar soils were selected under binoculars in a glovebox. Each grain was placed on a copper mesh of a customized ceramic plate and then placed into a sealed box while being contained in the glovebox prior to transfer and reflection infrared (IR) spectra measurements. The reflection IR spectra were measured under a high-purity nitrogen purging environment. This ensured that the lunar grains were not contaminated by terrestrial air in the transfer process and during the IR measurement. Prior to the NanoSIMS analyses, the lunar grains were embedded in indium discs in the glovebox and coated with Au with exposure to air for less than 10 min. The samples in our study were untreated and could be considered to have negligible terrestrial contamination. The smooth areas in the proximity of the points of NanoSIMS analyses of the grains were selected to prepare slices for transmission electron microscopy (TEM) and energy-dispersive X-ray spectroscopy (EDS) analyses. Eight mineral grains (two pyroxene, three plagioclase, and three olivine grains) with different values of water content resulting from solar wind implantation were selected for detailed studies. The chemical compositions of these lunar minerals are listed in Supplementary Table 2.

### Measurements of reflection infrared (IR) spectra

Grains with sizes in the range 78–272 μm were selected from CE5 lunar samples (CE5C0400YJFM00505) under binoculars in a glovebox with flowing high-purity argon. Reflection IR spectra of the untreated grains in the range 650–8000 cm^−1^ were collected using a Nicolet iS50 FTIR Spectrometer coupled with a Continuμm infrared (IR) microscope (Thermo Fisher Scientific) at the Institute of Geochemistry, Chinese Academy of Sciences. The IR microscope was enclosed in a custom-made glovebox and continuously purged with high-purity nitrogen throughout the measurement process. The samples were stored and transferred to a container filled with high-purity argon. This ensured that all the absorption peaks in the infrared spectra associated with water represented the characteristics of the lunar grains, and any possible interference resulting from terrestrial water was thus eliminated. Each reflection spectrum was based on data accumulated from 256 scans at a 4 cm^−1^ resolution, with the aperture size being 75 × 75 μm.

The OH/H_2_O content of the minerals was estimated using calibration lines derived from the analyses of three sets of terrestrial minerals that included olivine, plagioclase, and pyroxene grains with sizes varying from 100 to 200 μm (Supplementary Fig. 2). Reflection IR spectra and NanoSIMS analyses were conducted on terrestrial minerals using the same methods as used for lunar minerals. The calibration lines were obtained based on the depth of absorption bands in the range 3200–3800 cm^−1^ measured using FTIR and the water content calculated using NanoSIMS. To reduce the analytical errors, the calibration lines were deliberately made to pass through the origin of coordinates. The slopes of the calibration lines for olivine, plagioclase, and pyroxene were (6.07 ± 0.547) × 10^−4^, (3.12 ± 0.205) × 10^−4^, and (5.99 ± 0.838) × 10^−4^, respectively. The 2σ uncertainties of OH/H_2_O content derived from reflection IR spectra include errors in measurements conducted for terrestrial minerals and counting statistics of each analysis.

### NanoSIMS measurements

On the basis of preliminary identification of the composition and relative water content of the grains based on their reflection IR spectra, we selected several pyroxenes, plagioclase, and olivine grains with different values of water content. Measurements of water content and hydrogen isotope ratio in the lunar minerals were performed with a CAMECA NanoSIMS 50 L microprobe at the Institute of Geology and Geophysics, Chinese Academy of Sciences. Before NanoSIMS analysis, the lunar minerals were embedded in indium discs in a glovebox, and then, they were coated with Au and loaded in sample holders with a set of standers. Then, the holders were stored in the NanoSIMS sample chamber with the exposure of the minerals to air limited to less than 10 min. The vacuum for the chamber during analysis was <3.0 × 10^−10^ torr. A Cs^+^ ion beam current of 300 pA was used for the NanoSIMS measurements, and the secondary anions ^1^H^−^, ^2^D^−^, ^12^C^−^, and ^18^O^−^ were simultaneously collected on the electron multipliers (EMs) and a Faraday cup. Prior to analysis, the surface of samples was pre-sputtered for approximately 10 s on a 10 × 10 μm area to remove the Au coating and eliminate terrestrial contamination, which was monitored by the real-time imaging mode. Analysis was performed on the central 5 × 5 μm area using the blanking technique, and each measurement comprised 200 cycles. A 44 ns dead time was corrected for all EMs, and an electron gun was used for charge compensation. The spots chosen for the NanoSIMS analyses coincided with the areas of the reflection IR spectra measurements.

The measurements of NanoSIMS are the values of 200 cycles, which correspond to the measured depths of olivine, plagioclase, and pyroxene 264, 264, and 142 nm, respectively. For each measurement, 200 cycles of the ^1^H^−^, ^2^D^−^, and ^18^O^−^ were collected using the NanoSIMS. The total values of ^1^H^−^, ^2^D^−^, and ^18^O^−^ were calculated respectively for 200 cycles, and then the ratios of ^1^H^−^/^18^O^−^ and ^2^D^−^/^1^H^−^ were obtained. Thus, all reported water content and hydrogen isotope ratio in our study represents the average values for the entire 200 cycles (Supplementary Figs. 6–8, Supplementary Data 2). The water content of lunar minerals was determined using a ^1^H^−^/^18^O^−^ and H_2_O calibration line based on a set of terrestrial standards, including Kovdor apatite, basaltic glass 1838, SWIFT MORB glass, Durango apatite, and San Carlos olivine, for which the water content and δD values have been reported^22^. The slope of the calibration line is (7.149 ± 0.0639) × 10^−5^ (Supplementary Fig. 9). The ^1^H^−^/^18^O^−^ in anhydrous San Carlos olivine was used to assess the hydrogen background of NanoSIMS, corresponding to the water content of 12 ± 2.07 ppm. This value was subtracted from each of the water content values determined in the lunar minerals. The errors from the instrumental mass fractionation (IMF) factor were corrected by the analyses of the above standards. The average IMF factor for NanoSIMS analysis was calculated to be 1.05 ± 0.025. All reported values were corrected for the IMF. The measured D/H rations were expressed in terms of δD, δD = {[(D/H)_sample_/(D/H)_VSMOW_] − 1} × 1000‰, where D/H_VSMOW_ = 1.56 × 10^−4^. All values were reported with 2σ uncertainties, including the reproducibility of D/H measurements on the standards, uncertainty of H_2_O background subtraction, and internal precision. The water content and δD values were not corrected for the effects of cosmic-ray spallation, as the correction has virtually no effect on values obtained by analysis of samples with high water content^26^.

### TEM and EDS analyses

After the NanoSIMS measurements, a focused ion beam scanning electron microscope (FIB-SEM) system was used to determine the morphology and prepare slices of the surface (~5 μm) of pyroxene, plagioclase, and olivine grains for TEM analyses at the Institute of Geochemistry, Chinese Academy of Sciences. The positions of the slices were chosen close to the NanoSIMS measurement points and in the area of reflection IR spectra measurements. TEM analyses involving bright and dark-field imaging of the slices were conducted using a Talos F200S TEM (Thermo Fisher Scientific) at Guangdong University of Technology. The chemical composition was determined by means of the EDS installed in the TEM. The composition profiles determined by EDS were used to analyze the modification process that the samples experienced. The damaged rims exhibited a chemical composition consistent with that of the underlying crystals, except for a slight decrease in oxygen content (Supplementary Figs. 3–5). This indicates that the damaged rims were produced by the implantation of solar wind particles, which could cause oxygen sputtering. The impacts of meteorite and micrometeorite are also important processes on the lunar surface. However, the rims formed from vapor deposition following meteoroid impacts can be distinguished by TEM and EDS results, which exhibit a significant difference in composition from the underlying layers^45^. Even small amounts of insignificant vapor deposition in the reflection IR and NanoSIMS measurement areas do not affect the results of water content. Zhu et al. demonstrated^46^ that OH/H_2_O formed in silicates by implantation of solar wind H^+^ could be released into the gas phase after micrometeorite impact. Meteorite and micrometeorite impacts would reduce the content of solar wind-derived OH/H_2_O. Considering the conditions of ultra-high vacuum and the high temperature caused by impact, the hydrogen isotopes undergo fractionation and result in relatively obvious changes in the δD value of water in the rims. From the δD analysis in this study, this effect is negligible. Based on the results, eight minerals (two pyroxene, three plagioclase, and three olivine grains) without visible characteristics of micrometeorite impacts and high-temperature melting were chosen for detailed analyses.

### Comparison of water content determined by NanoSIMS and reflection IR

The water content obtained from NanoSIMS is much higher than that from reflection IR, which is mainly attributed to the difference in measurement depth. The NanoSIMS analysis obtained the concentration of water at a depth of ~200 nm on the upper surface of mineral grains, whereas the reflectance IR determined the concentration at the micron-level depth. The water derived from the solar wind is mainly retained within a few hundred nanometers of the grain surface; thus, the water content observed from NanoSIMS and reflection IR exhibited significant differences. However, there are three main issues that complicate the comparison of NanoSIMS and reflection IR results. First, the exact penetration depth of the reflection IR is hardly determined. Second, solar wind-implanted H^+^ diffuses inward to deeper locations than the damaged rim, and this diffusion depth depends on the surface temperature and activation energy of each grain. Third, the different occurrences of H measured by NanoSIMS and reflection IR can cause some differences in the results between NanoSIMS and reflection IR.

Based on the zeroth-order incoherent microwave radiative transfer model, Lv et al. deduced^47^ the penetration depth (D) of the soil at different wavelengths (*λ*) with the following equation:

1 $${{{{{\rm{D}}}}}}=\frac{\lambda}{2\Pi }\,.\,\frac{\sqrt{{\varepsilon} ^{\prime}}}{\varepsilon^{{\prime} {\prime}} }$$

where $\sqrt{{\varepsilon}^{\prime} }$ and $\varepsilon^{{\prime} {\prime}}$ are the real part and imaginary part of the dielectric constant. The mean dielectric permittivity of the lunar regolith has been estimated to be 2.96 + i0.03 based on its composition and porosity^43^. Using this parameter, although the uncertainty is large, we estimated a penetration depth of ~26 µm for the 2.85 µm wavelength. In addition, the sampling depth of the ATR method is approximately 2–15 µm, is wavelength dependent, and increases with decreasing wavenumber^48^. We assume that the penetration depth of infrared IR in this study is from 3 to 30 µm. The water content determined by NanoSIMS in the ~200 nm depth was converted to 3–30 µm, as shown in Supplementary Table 4. The water content of grains converted from the NanoSIMS results greatly varies depending on the penetration depth. It seems that the NanoSIMS results relatively match the reflection IR results when the penetration depth is approximately similar to the wavelength (3 µm).

### Calculation of solar wind-derived water content and the corresponding exposure time of the CE5 lunar soils

With the assumption that all the water is formed in olivine, plagioclase, and pyroxene via the interaction of solar wind protons with soils of the lunar surface, the solar wind-derived water content in CE5 lunar soils can be estimated using the reflectance IR spectra. CE5 lunar soils contain ~5.7% olivine, ~30.1% plagioclase, and ~42% pyroxene. Taking together the values of the mineral abundance of CE5 lunar soils and the average water content calculated based on reflectance IR spectra of lunar minerals (Supplementary Table 1), the water content was estimated to be ~170 ppm in CE5 lunar soils. Additionally, agglutinates and volcanic glasses are known to retain large amounts of solar wind implantation^8,10^. Therefore, the overall quantity of water generated by the solar wind in CE5 lunar soils is expected to be larger.

The lunar grains in our study could have undergone a complex escape process, which would reduce the amount of initial solar wind-derived water in the minerals. As the heating experiment showed that the content of solar wind-derived water formed decreased with increasing lunar surface temperature, it was estimated that ~24% of the amount of water was lost, giving a maximum temperature of ~350 K in the CE5 region^13,42^. Considering only the effect of temperature on water content, the initial water content on the surface (~200 nm) of lunar minerals was 1205–7845 ppm. Previous studies have suggested that ~20–50% of solar wind protons can be bonded with oxygen to form OH/H_2_O^49^. Based on this, we calculated the surface density of hydrogen (*ρ*_*H*_) from the following equation^50^:

2 $${{{{{{\rm{H}}}}}}}_{2}{{{{{\rm{O}}}}}}({{{{{\rm{wt}}}}}}\%)=\left(\,\frac{p\,.\,{\rho }_{H}}{2{{{{{\rm{D}}}}}}}/{{{{{{\rm{N}}}}}}}_{{{{{{\rm{A}}}}}}}\right)\,.\,18/{\rho }_{m}$$

where the *p* is the fraction of hydrogen bonded to oxygen, and the value of 20–50% was taken for our calculations. D is the penetration depth for solar wind protons, N_A_ is the Avogadro’s constant (6.02 × 10^23^), and *ρ*_*m*_ is the density of minerals, which is 3.35 g/cm^3^ for olivine, 2.73 g/cm^3^ for plagioclase, and 3.21 g/cm^3^ for pyroxene. Combined with the proton flux of solar wind (4 × 10^15^ H/cm^−2^/yr), we calculated the accumulated implantation time of solar wind for lunar minerals to be ~270–4212 years (Supplementary Table 5). However, there may be considerable uncertainty in these values, such as the conversion of proton flux to OH/H_2_O, for which values can vary considerably^15,50^, the complex factors influencing the formation processes of solar wind-derived water, the escape process of solar wind-derived water by temperature and ultraviolet radiation, the diffusion of solar wind-derived water. Estimation of the exposure age of solar wind implantation for lunar soil still requires further investigation.

### Deriving the ESPAT values and corresponding water content from reflection infrared spectra

To quantify the water content in the lunar regolith by ESPAT values, the reflection spectra were first converted into single-scattering albedo (SSA) spectra based on the Hapke radiative transfer model. For single grains in our study, this method is not applicable. According to the single particle empirical formula, we converted the reflection spectra of mineral grains into *S*_*e*_ spectra (*ω*) using the following equation^40^:

3 $${S}_{e}=0.0587+0.8543R(0)+{0.0870R(0)}^{2}$$

where *R*(0) is the reflectivity at a zero angle of incidence. Then, the *S*_*e*_ spectra between 2.5 and 3.3 µm were continuum-removed to determine the ESPAT number for the water absorptions at the wavelength of 2.85–3 µm:

4 $${{{{{\rm{ESPAT}}}}}}=\frac{1-\varpi }{\varpi }$$

where *ϖ* is the *ω* at the maximum absorption position between 2.85 and 3 µm in the *S*_*e*_ spectra after continuum removal. The corresponding water content was calculated by multiplying the slopes deduced from the previous simulation^6^. Our calculations show that these ESPAT values are generally higher than those derived from remote sensing data; however, the water content calculated from ESPAT values is consistent with that determined by reflection infrared spectra (Supplementary Table 3). The difference is mainly attributed to the size of grains, as our grains were hand-picked from CE5 samples with large grain sizes.

## Supplementary information

Supplementary Information

Description of Additional Supplementary Files

Supplementary Data 1

Supplementary Data 2

## Data Availability

All data are available in the main text or the supplementary information. The reflectance IR and NanoSIMS data generated in this study are provided in the Source Data file. The original SEM, TEM, EDS data, and original image of OMAT image used in this study are available in the Mendeley Data database under [https://data.mendeley.com/datasets/fcwyz3kv3k/1]^51^.

## References

[1] Clark RN (2009). Detection of adsorbed water and hydroxyl on the Moon. Science.

[2] Sunshine JM (2009). Temporal and spatial variability of lunar hydration as observed by the Deep Impact spacecraft. Science.

[3] Pieters CM (2009). Character and spatial distribution of OH/H_2_O on the surface of the Moon seen by M3 on Chandrayaan-1. Science.

[4] Bandfield JL, Poston MJ, Klima RL, Edwards CSJNG (2018). Widespread distribution of OH/H_2_O on the lunar surface inferred from spectral data. Nat. Geosci..

[5] McCord, T. et al. Sources and physical processes responsible for OH/H_2_O in the lunar soil as revealed by the Moon Mineralogy Mapper (M3). *J. Geophys. Res*. **116**, E00G05 (2011).

[6] Li S, Milliken RE (2017). Water on the surface of the Moon as seen by the Moon Mineralogy Mapper: Distribution, abundance, and origins. Sci. Adv..

[7] Wöhler C, Grumpe A, Berezhnoy AA, Shevchenko VV (2017). Time-of-day–dependent global distribution of lunar surficial water/hydroxyl. Sci. Adv..

[8] Liu Y (2012). Direct measurement of hydroxyl in the lunar regolith and the origin of lunar surface water. Nat. Geosci..

[9] Izawa MR (2014). Laboratory spectroscopic detection of hydration in pristine lunar regolith. Earth Planet. Sci. Lett..

[10] Stephant A, Robert F (2014). The negligible chondritic contribution in the lunar soils water. PNAS.

[11] Managadze GG, Cherepin VT, Shkuratov Y, Kolesnik V, Chumikov A (2011). Simulating OH/H_2_O formation by solar wind at the lunar surface. Icarus.

[12] Bradley JP (2014). Detection of solar wind-produced water in irradiated rims on silicate minerals. PNAS.

[13] Zeng X (2021). Experimental investigation of OH/H_2_O in H^+^-irradiated plagioclase: implications for the thermal stability of water on the lunar surface. Earth Planet. Sci. Lett..

[14] Schaible MJ, Baragiola RA (2014). Hydrogen implantation in silicates: the role of solar wind in SiOH bond formation on the surfaces of airless bodies in space. J. Geophys. Res..

[15] Tang H (2021). Experimental investigation of structural OH/H_2_O in different lunar minerals and glass via solar-wind proton implantation. Icarus.

[16] Qian Y (2021). China’s Chang’e-5 landing site: geology, stratigraphy, and provenance of materials. Earth Planet. Sci. Lett..

[17] Tian H (2021). Non-KREEP origin for Chang’e-5 basalts in the Procellarum KREEP Terrane. Nature.

[18] Li Q (2021). Two-billion-year-old volcanism on the Moon from Chang’e-5 basalts. Nature.

[19] Greenwood JP (2011). Hydrogen isotope ratios in lunar rocks indicate delivery of cometary water to the Moon. Nat. Geosci..

[20] Anand M, Tartèse R, Barnes JJ (2014). Understanding the origin and evolution of water in the Moon through lunar sample studies. Philos. Trans. R. Soc. A.

[21] Černok A (2020). Preservation of primordial signatures of water in highly-shocked ancient lunar rocks. Earth Planet. Sci. Lett..

[22] Hu, S. et al. A dry lunar mantle reservoir for young mare basalts of Chang’e-5. *Nature* **600**, 49–53 (2021). 10.1038/s41586-021-04107-9 PMC8636271 34666337

[23] Li C (2022). Characteristics of the lunar samples returned by the Chang’E-5 mission. Natl Sci. Rev..

[24] Honniball C (2021). Molecular water detected on the sunlit Moon by SOFIA. Nat. Astron..

[25] Johnson EA, Rossman GR (2004). A survey of hydrous species and concentrations in igneous feldspars. Am. Mineral..

[26] Füri E, Deloule E, Trappitsch R (2017). The production rate of cosmogenic deuterium at the Moon’s surface. Earth Planet. Sci. Lett..

[27] Füri E, Zimmermann L, Deloule E, Trappitsch R (2020). Cosmic ray effects on the isotope composition of hydrogen and noble gases in lunar samples: insights from Apollo 12018. Earth Planet. Sci. Lett..

[28] Keller LP, Berger EL, Zhang S, Christoffersen R (2021). Solar energetic particle tracks in lunar samples: a transmission electron microscope calibration and implications for lunar space weathering. Meteorit. Planet Sci..

[29] Hapke B (2001). Space weathering from Mercury to the asteroid belt. J. Geophys. Res..

[30] Kuhlman KR, Sridharan K, Kvit A (2015). Simulation of solar wind space weathering in orthopyroxene. Planet. Space Sci..

[31] Gu L (2022). Space weathering of the Chang’e-5 lunar sample from a mid-high latitude region on the Moon. Geophys. Res. Lett..

[32] Keller, L. & Zhang, S. Rates of space weathering in lunar soils. In *Space Weathering of Airless Bodies: An Integration of Remote Sensing Data, Laboratory Experiments and Sample Analysis Workshop*, vol. 1878, 2056 (LPI, 2015).

[33] Poppe A, Farrell W, Halekas JS (2018). Formation timescales of amorphous rims on lunar grains derived from ARTEMIS observations. J. Geophys. Res..

[34] Wang, L. & Ewing, R. Ion beam-induced amorphization of (Mg, Fe) _2_SiO_4_ olivine series: an in situ transmission electron microscopy study. *Mater. Res. Soc. Symp. Proc*. **235**, 333–338 (1991).

[35] Jäger C (2003). Structural processing of enstatite by ion bombardment. Astron. Astrophys..

[36] Li Y (2013). Crystal orientation results in different amorphization of olivine during solar wind implantation. J. Geophys. Res..

[37] Lin H (2022). In situ detection of water on the Moon by the Chang’E-5 lander. Sci. Adv..

[38] Liu J (2022). Evidence of water on the lunar surface from Chang’E-5 in-situ spectra and returned samples. Nat. Commun..

[39] Milliken RE, Li S (2017). Remote detection of widespread indigenous water in lunar pyroclastic deposits. Nat. Geosci..

[40] Hapke, B. *Theory of Reflectance and Emittance Spectroscopy*. (Cambridge university press, 2012).

[41] Lucey PG, Blewett DT, Taylor GJ, Hawke BR (2000). Imaging of lunar surface maturity. J. Geophys. Res..

[42] Williams J-P, Paige D, Greenhagen B, Sefton-Nash E (2017). The global surface temperatures of the Moon as measured by the Diviner Lunar Radiometer Experiment. Icarus.

[43] Jia B, Fa W, Xie M, Tai Y, Liu X (2021). Regolith properties in the Chang’E-5 landing region of the Moon: results from multi-source remote sensing observations. J. Geophys. Res..

[44] Lemelin M (2019). The compositions of the lunar crust and upper mantle: spectral analysis of the inner rings of lunar impact basins. Planet. Space Sci..

[45] Guo Z (2022). Nanophase iron particles derived from fayalitic olivine decomposition in Chang’E-5 lunar soil: implications for thermal effects during impacts. Geophys. Res. Lett..

[46] Zhu C (2019). Untangling the formation and liberation of water in the lunar regolith. PNAS.

[47] Lv S, Zeng Y, Wen J, Zhao H, Su Z (2018). Estimation of penetration depth from soil effective temperature in microwave radiometry. Remote Sens..

[48] Larkin, P. *Infrared and Raman Spectroscopy: Principles and Spectral Interpretation*. (Elsevier, 2017).

[49] Epstein, S. & Taylor Jr, H. O^18^/O^16^, Si^30^/Si^28^, C^13^/C^12^, and D/H studies of Apollo 14 and 15 samples. In *Proceedings of the Third Lunar Science Conference*, Vol. 3, 1429 (MIT Press, 1972).

[50] McLain J (2021). Hydroxylation of Apollo 17 soil sample 78421 by solar wind protons. J. Geophys. Res..

[51] Zhou, C. Original data for Chang’E-5 samples reveal high water content in lunar minerals. *Mendeley Data*10.17632/fcwyz3kv3k.1 (2022). 10.1038/s41467-022-33095-1 PMC9464205 36088436

