# Supplementary material for: Chang’E-5 samples reveal high water content in lunar minerals
Source: Nat Commun. 2022 Sep 10;13:5336. doi: 10.1038/s41467-022-33095-1 (PMC9464205; doi:10.1038/s41467-022-33095-1)
Supplement: Supplementary file 1 — Supplementary Information [file 41467_2022_33095_MOESM1_ESM.pdf]

Supplementary information for

Chang'E-5 samples reveal high water content in lunar minerals

Chuanjiao Zhou[1,2], Hong Tang[1,3,4,*], Xiongyao Li[1,3,4,*], Xiaojia Zeng[1,4], Bing Mo[1,3,4], Wen Yu[1,3,4], Yanxue Wu[5], Xiandi Zeng[1,2], Jianzhong Liu[1,3,4], Yuanyun Wen[1]

[1]Center for Lunar and Planetary Sciences, Institute of Geochemistry, Chinese Academy of Sciences, Guiyang 550081, China.

[2]College of Earth and Planetary Sciences, University of Chinese Academy of Sciences, Beijing 100049, China.

[3]CAS Center for Excellence in Comparative Planetology, Hefei 230026, China.

[4]Key Laboratory of Space Manufacturing Technology, Chinese Academy of Sciences, Beijing 100094, China.

[5]Analysis and Test Center, Guangdong University of Technology, Guangzhou 510006, China.

*Corresponding Author. E-mail address: tanghong@vip.gyig.ac.cn; lixiongyao@vip.skleg.cn

The PDF file includes:

Supplementary Figures

Supplementary Fig. 1. Reflectance IR spectra of CE-PL2 lunar grains in the region of 1,900–1,400 cm^{-1}.

Supplementary Fig. 2. Calibration lines based on OH/H_2O content of terrestrial minerals.

Supplementary Fig. 3. High-angle annular dark-field (HAADF) images and corresponding composition profiles of olivine samples.

Supplementary Fig. 4. High-angle annular dark-field (HAADF) images and corresponding composition profiles of plagioclase samples.

Supplementary Fig. 5. High-angle annular dark-field (HAADF) images and corresponding composition profiles of pyroxene samples.

Supplementary Fig. 6. Results of NanoSIMS analysis of CE5 olivine grains.

Supplementary Fig. 7. NanoSIMS analysis results of CE5 plagioclase grains.

Supplementary Fig. 8. Results of NanoSIMS analysis of CE5 pyroxene grains.

Supplementary Fig. 9. Calibration line for NanoSIMS analysis of water content in standards.

Supplementary Tables

Supplementary Table 1. The OH/H_2O content determined by reflectance spectra of lunar grains.

Supplementary Table 2. Composition and NanoSIMS analytical results of lunar grains.

Supplementary Table 3. ESPAT values with the corresponding water content calculated from reflection infrared spectra of mineral grains**.**

Supplementary Table 4. Comparison of water content converted from NanoSIMS results to 3–30 μm and determined by FTIR results.

Supplementary Table 5. Accumulated implantation time of solar wind for lunar minerals.

Supplementary Figures

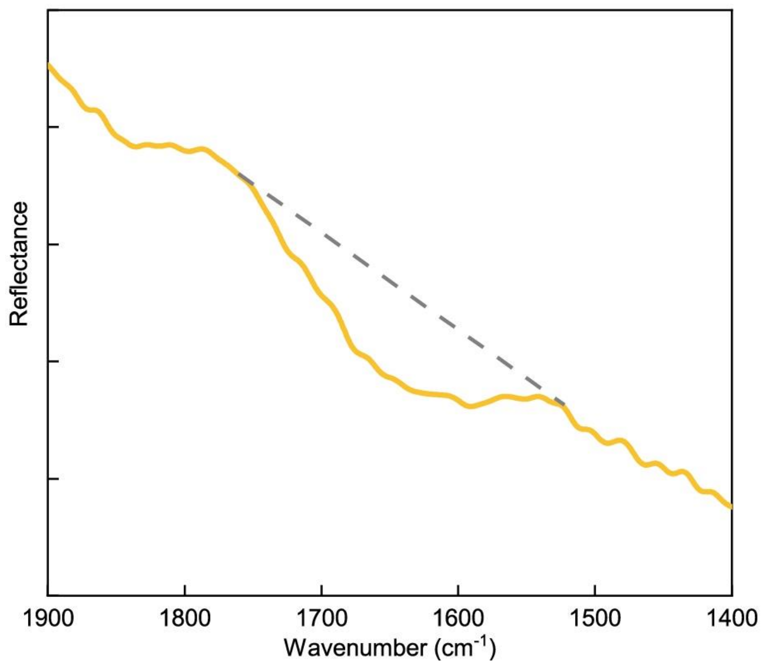

Supplementary Figure 1. Reflectance IR spectra of CE-PL2 lunar grains in the region of 1,900–1,400 cm^{-1}. The solid lines are smoothed spectra obtained using the Fast Fourier Transform algorithm; the dashed lines indicate the baseline reflectance of the absorption peaks.

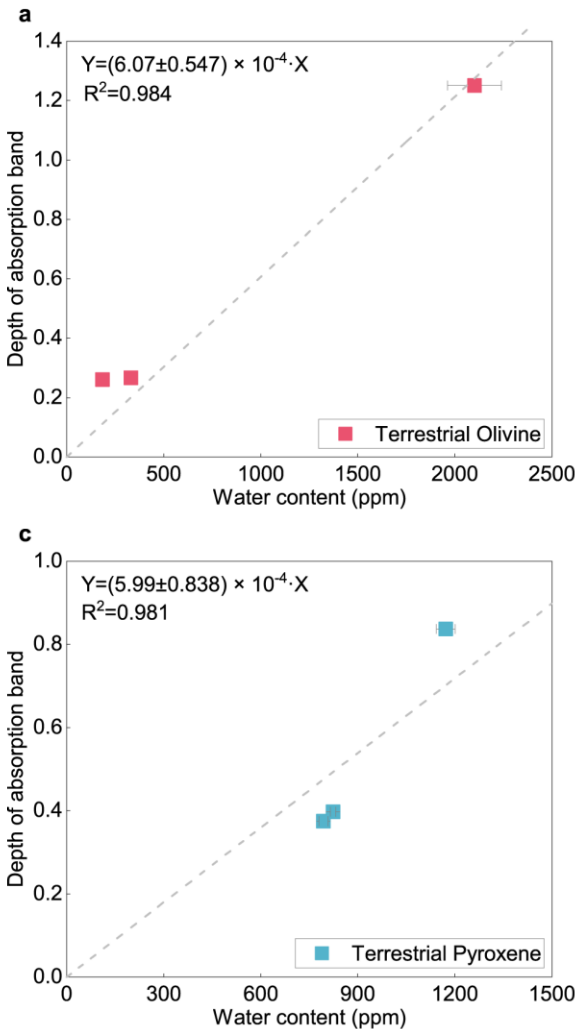

Supplementary Figure 2. Calibration lines based on OH/H_2O content of terrestrial minerals. **a-c** Calibration lines derived from the depth of absorption band measured by reflectance IR and water content measured by NanoSIMS for **a** Terrestrial Olivine, **b** Terrestrial Plagioclase, and **c** Terrestrial Pyroxene, respectively. The slopes of the calibration line for terrestrial olivine, terrestrial plagioclase, and terrestrial pyroxene are $(6.07 \pm 0.547) \times 10^{-4}$, $(3.12 \pm 0.205) \times 10^{-4}$, and $(5.99 \pm 0.838) \times 10^{-4}$, respectively. The coefficients of determination for calibration line of terrestrial olivine, terrestrial plagioclase, and terrestrial pyroxene are 0.984, 0.991, and 0.981, respectively. The error bars represent 2σ.

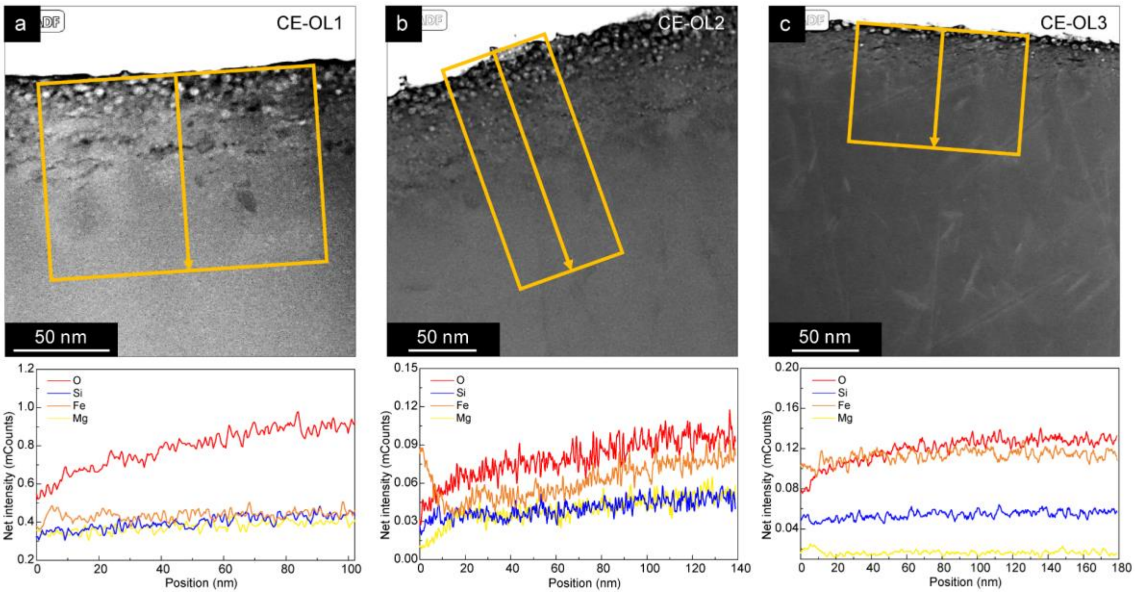

Supplementary Figure 3. High-angle annular dark-field (HAADF) images and corresponding composition profiles of olivine samples. a-c The HAADF images (top) and composition profiles (bottom) in orange boxes of **a** CE-OL1, **b** CE-OL2, **c** CE-OL3. The scale bar of HAADF images is 50 nm.

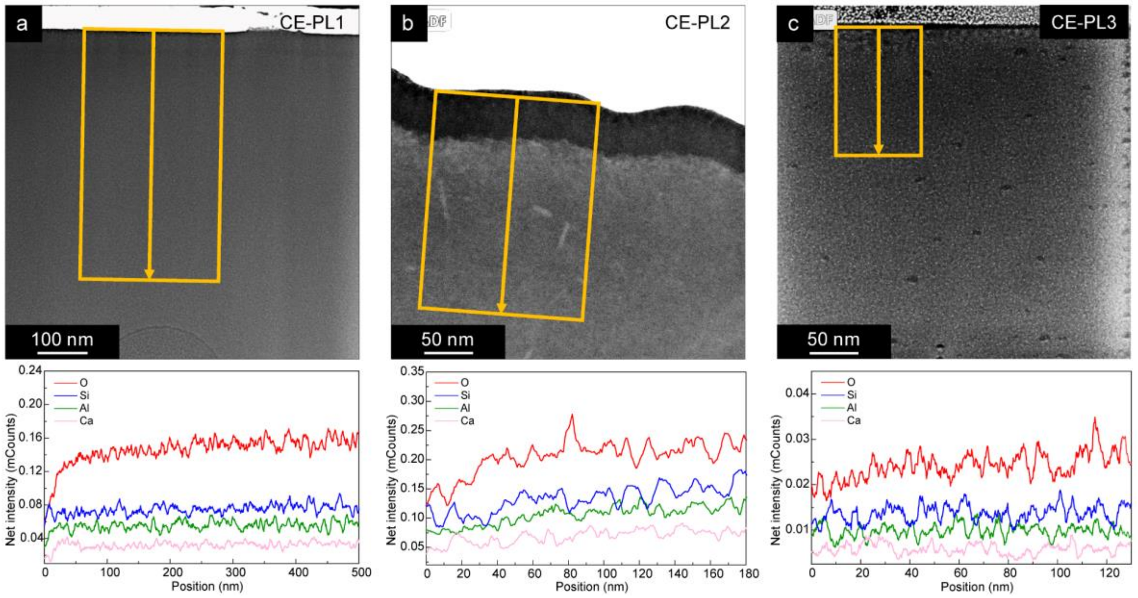

Supplementary Figure 4. High-angle annular dark-field (HAADF) images and corresponding composition profiles of plagioclase samples. a-c The HAADF images (top) and composition profiles (bottom) in orange boxes of **a** CE-PL1, **b** CE-PL2, **c** CE-PL3. The scale bar of HAADF images is 100 nm for CE-PL1 and 50 nm for CE-PL2 and CE-PL3.

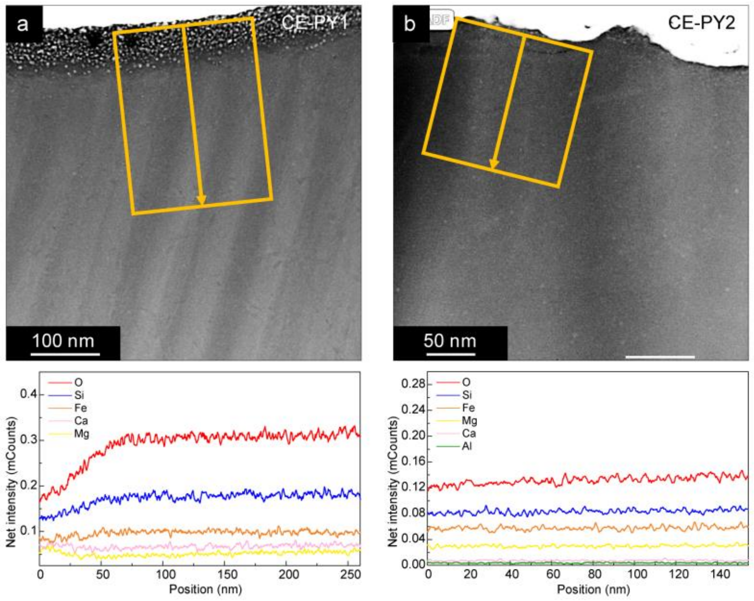

Supplementary Figure 5. High-angle annular dark-field (HAADF) images and corresponding composition profiles of pyroxene samples. a, b The HAADF images (top) and composition profiles (bottom) in orange boxes of **a** CE-PY1, **b** CE-PY2. The scale bar of HAADF images is 100 nm for CE-PY1 and 50 nm for CE-PY2.

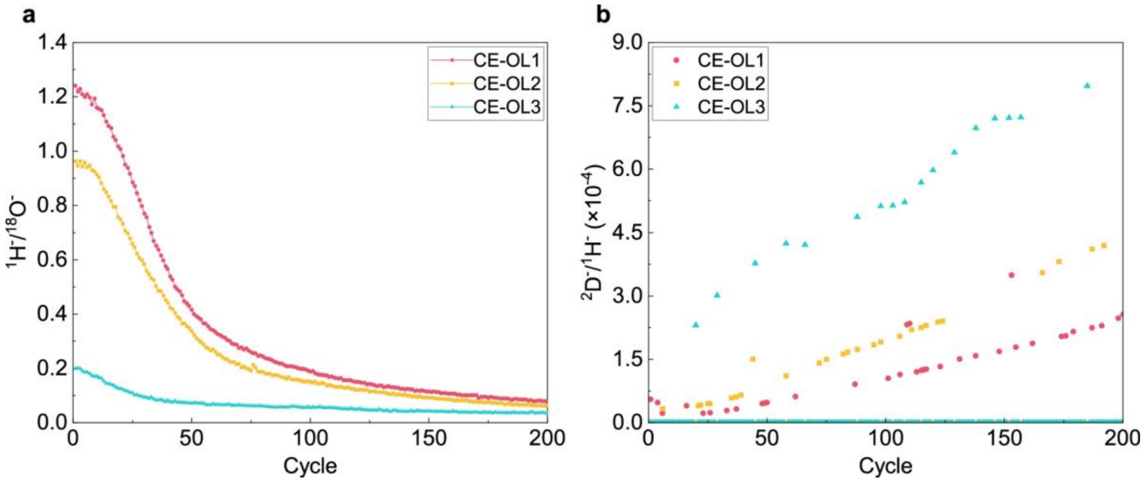

Supplementary Figure 6. Results of NanoSIMS analysis of CE5 olivine grains. a $^1H^-/^{18}O^-$ and **b** $^2D^-/^1H^-$ results of olivine samples collected by NanoSIMS.

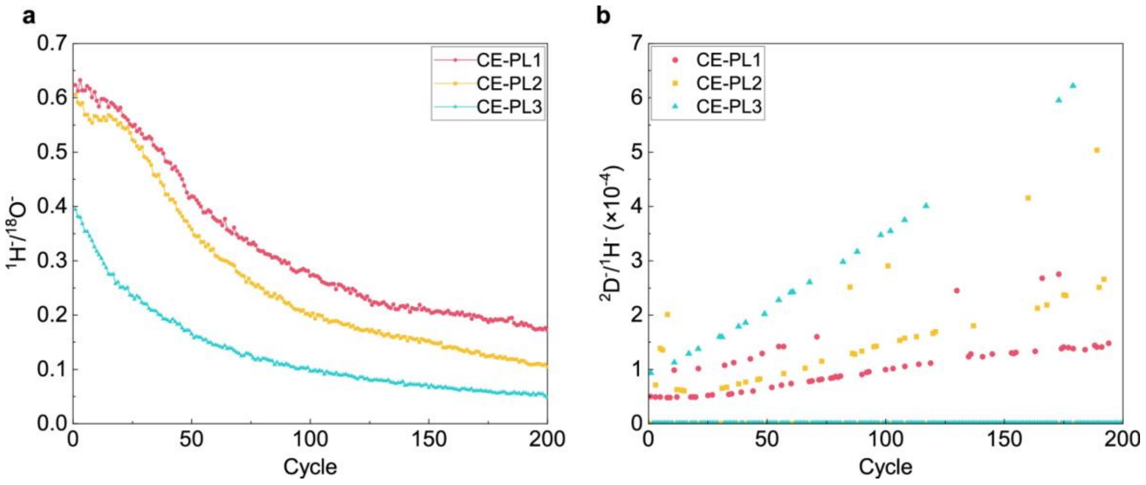

Supplementary Figure 7. NanoSIMS analysis results of CE5 plagioclase grains. a $^1H^-/^{18}O^-$ and **b** $^2D^-/^1H^-$ results of plagioclase samples collected by NanoSIMS.

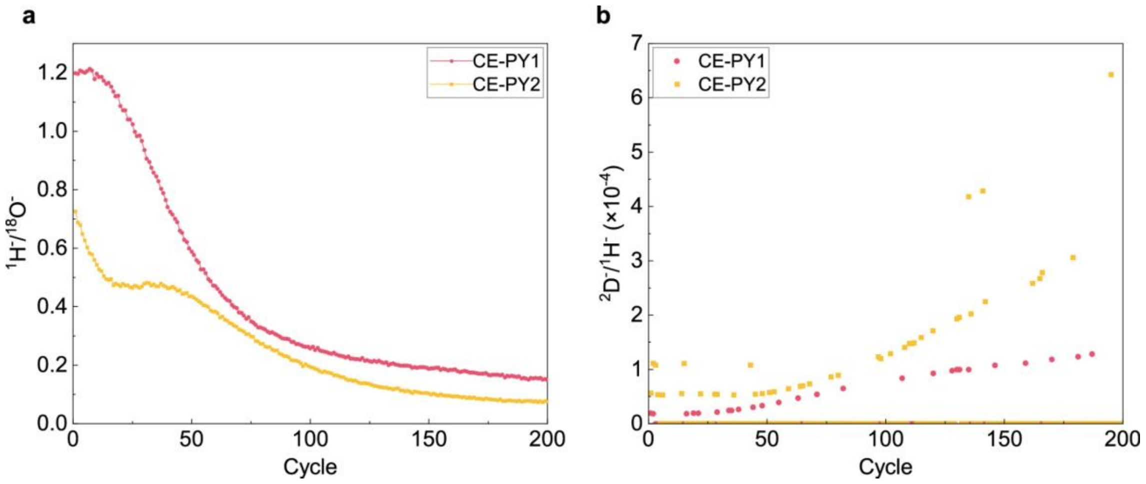

Supplementary Figure 8. Results of NanoSIMS analysis of CE5 pyroxene grains. a $^1H^-/^{18}O^-$ and **b** $^2D^-/^1H^-$ results of pyroxene samples collected by NanoSIMS.

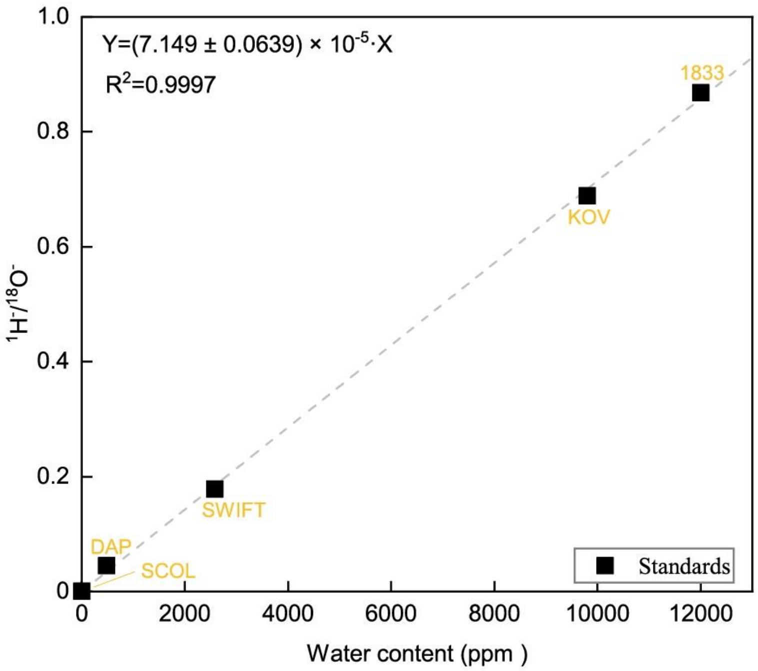

Supplementary Figure 9. Calibration line for NanoSIMS analysis of water content in standards. The slope of the calibration line is $(7.149 \pm 0.0639) \times 10^{-5}$ with the coefficient of determination of 0.9997. SCOL: San Carlos olivine, DAP: Durango apatite, SWIFT: SWIFT MORB glass (N = 24); KOV: Kovdor apatite, 1833: basaltic glass 1833. The analytical uncertainty is 0.50 %.

Supplementary Tables

Supplementary Table 1. The OH/H_2O content determined by reflectance spectra of lunar grains. The depth of 3,200–3,800 cm^{-1} absorption bands from reflectance infrared spectra, which represents the existence of OH/H_2O.

Sample	Depth of OH/H_2O absorption band	OH/H_2O content (ppm)	Average OH/H_2O content (ppm)
Olivine			209 ± 20
CE-OL1	0.189	311 ± 30	
CE-OL2	0.100	165 ± 16	
CE-OL3	0.092	152 ± 14	
Plagioclase			295 ± 20
CE-PL1	0.120	385 ± 27	
CE-PL2	0.072	231 ± 16	
CE-PL3	0.084	269 ± 19	
Pyroxene			166 ± 23
CE-PY1	0.080	134 ± 19	
CE-PY2	0.119	199 ± 28	

Supplementary Table 2. Composition and NanoSIMS analytical results of lunar grains. The compositions of pyroxene were identified by selected area electron diffraction (SAED) results.

Sample	Composition	$^{1}H^{-}/^{18}O^{-}$	Water content (ppm) ± 2σ	$^{2}D^{-}/^{1}H^{-}$ ± 2σ	δD (‰) ± 2σ
CE-OL1	Fo[1] = 61	3.21×10^{-1}	4483 ± 314	1.29×10^{-5}	-921 ± 174
CE-OL2	Fo = 52	2.61×10^{-1}	3632 ± 251	1.57×10^{-5}	-904 ± 198
CE-OL3	Fo = 19	6.63×10^{-2}	916 ± 38	3.71×10^{-5}	-773 ± 188
CE-PLI	An[2] = 76	3.21×10^{-1}	4476 ± 142	3.24×10^{-5}	-802 ± 97
CE-PL2	An = 84	2.57×10^{-1}	3578 ± 147	2.66×10^{-5}	-837 ± 150
CE-PL3	An = 83	1.29×10^{-1}	1798 ± 81	2.84×10^{-5}	-827 ± 196
CE-PY1	Augite	4.27×10^{-1}	5962 ± 335	8.98×10^{-6}	-945 ± 384
CE-PY2	Hypersthene and Augite[3]	2.49×10^{-1}	3471 ± 166	2.45×10^{-5}	-850 ± 160

[1]Fo = 100 × Mg/[Mg + Fe] molar ratio
[2]An = 100 × Ca/[Ca + Na + K] molar ratio
[3]The CE-PY2 is characterized by the intergrowth of hypersthene and augite, which was observed by transmission electron microscopy (TEM).

Supplementary Table 3. ESPAT values with the corresponding water content calculated from reflection infrared spectra of mineral grains.

Sample	ESPAT value	Grain size (μm)	Slope	Water content calculated from ESPAT (ppm)
CE-OL1	0.046	103	5000	230
CE-OL2	0.045	215	3400	153
CE-OL3	0.056	272	3400	191
CE-PL1	0.052	241	3400	175
CE-PL2	0.028	78	5000	138
CE-PL3	0.080	220	3400	273
CE-PY1	0.018	93	5000	88
CE-PY2	0.081	228	3400	274

Supplementary Table 4. Comparison of water content converted from NanoSIMS results to 3–30 μm and determined by FTIR results. The depth of NanoSIMS analysis were obtained from the measurements of the NanoSIMS analysis slices prepared by the FIB-SEM system.

Sample	Depth of NanoSIMS analysis (nm)	Water content converted from NanoSIMS (ppm)	Water content determined by FTIR (ppm)
CE-OL1	264	39–395	311 ± 30
CE-OL2	264	32–320	165 ± 16
CE-OL3	264	8–81	152 ± 14
CE-PL1	264	39–394	385 ± 27
CE-PL2	264	31–315	231 ± 16
CE-PL3	264	16–158	269 ± 19
CE-PY1	142	28–282	134 ± 19
CE-PY2	142	16–164	199 ± 28

Supplementary Table 5. Accumulated implantation time of solar wind for lunar minerals.

Sample	Implantation time (year) p^1 = 20%	Implantation time (year) p = 50%
CE-OL1	3306	1322
CE-OL2	2678	1071
CE-OL3	675	270
CE-PL1	2690	1076
CE-PL2	2150	860
CE-PL3	1080	432
CE-PY1	4212	1685
CE-PY2	2452	981

[1]*p* is the fraction of hydrogen bonded with oxygen
