# Supplementary material for: Chang’E-5 samples reveal high water content in lunar minerals
Source: Nat Commun. 2022 Sep 10;13:5336. doi: 10.1038/s41467-022-33095-1 (PMC9464205; doi:10.1038/s41467-022-33095-1)
Supplement: Supplementary file 2 — Description of Additional Supplementary Files [file 41467_2022_33095_MOESM2_ESM.pdf]

File name: Supplementary Data 1

Description: The reflectance infrared spectra data of lunar mineral grains in the range 3,000–4,000 cm^{-1}.

File name: Supplementary Data 2

Description: The NanoSIMS data of lunar mineral grains, including the counts of hydrogen (^{1}H), deuterium (^{2}H), and oxygen (^{18}O).
